# Multiple introductions and onward transmission of non-pandemic HIV-1 subtype B strains in North America and Europe.

**DOI:** 10.1038/srep33971

**Published:** 2016-09-22

**Authors:** Marina Cabello, Hector Romero, Gonzalo Bello

**Affiliations:** 1 Laboratório de AIDS e Imunologia Molecular, Instituto Oswaldo Cruz, FIOCRUZ, Rio de Janeiro, Brazil; 2 Laboratorio de Organizacion y Evolucion del Genoma, Departamento de Ecologıa y Evolucion, Facultad de Ciencias-CURE, Universidad de la Republica, Montevideo, Uruguay

## Abstract

Most HIV-1 subtype B infections in North America and Europe seem to have resulted from the expansion of a single pandemic lineage (B_PANDEMIC_) disseminated from the United States (US). Some non-pandemic subtype B strains of Caribbean origin (B_CAR_) may have also reached North America and Europe, but their epidemiological relevance in those regions remains largely unknown. Here we analyze a total of 20,045 HIV-1 subtype B *pol* sequences from the US, Canada, and Europe, to estimate the prevalence and to reconstruct the spatiotemporal dynamics of dissemination of HIV-1 B_CAR_ strains in those regions. We find that B_CAR_ strains were probably disseminated from the Caribbean into North America and Europe at multiple times since the early 1970s onwards. The B_CAR_ strains reached the US, Canada and at least 16 different European countries, where they account for a very low fraction (<5%) of subtype B infections, with exception of the Czech Republic (7.7%). We also find evidence of the onward transmission of B_CAR_ clades in the US, Canada, the Czech Republic, Germany, Italy, Spain and the UK, as well as short-distance spreading of B_CAR_ lineages between neighboring European countries from Central and Western Europe, and long-distance dissemination between the US and Europe.

By the end of 2012, the total number of people living with the Human Immunodeficiency Virus Type 1 (HIV-1) reached an estimated 1.3 million [980.000–1.900.000] in North America and 860.000 [800.000–930.000] in Western/Central Europe^1^. Unprotected sex between men who have sex with men (MSM) is the main driver of HIV transmission in the US, Canada and Europe, followed by heterosexual contact and injecting drug use (IDU)^2^,^3^,^4^. The proportion of newly diagnosed HIV cases attributed to MSM increased in the US, Canada and Europe over the last 10 years, while the corresponding proportions attributed to heterosexual contact and IDU remained stable or decreased in the same period^2^,^3^,^4^.

Subtype B dominates the HIV-1 epidemic in North America and in Western and Central Europe^5^; although each country has a unique HIV molecular pattern and an increasing prevalence of non-B subtypes have been observed in the United States (US)^6^, Canada^7^, and Europe^8^,^9^,^10^,^11^ in the last decade. The HIV-1 subtype B epidemic in North America and Europe probably started via a single viral introduction from Haiti into the US around the late 1960s, followed by rapid viral dissemination within the US and from the US to other American countries and Europe, leading to the origin of a pandemic subtype B clade (B_PANDEMIC_)^12^. Networks of MSM and IDUs probably played a crucial role in the early spread of the B_PANDEMIC_ lineage within North America and from North America to Europe^13^,^14^,^15^. The HIV-1 subtype B epidemic in most European countries originated from multiple sources^16^,^17^, revealing the importance of human mobility across international borders in the spread of this clade. Current subtype B transmissions in Europe, however, seem to be predominantly occurring between patients from the same country^18^.

The study of Gilbert *et al*.^12^ also pointed to the existence of non-pandemic subtype B lineages that, in sharp contrast to the B_PANDEMIC_ clade, remained mostly restricted to the Caribbean region (B_CAR_ clades). More recent studies conducted by our group demonstrate that several non-pandemic B_CAR_ lineages have been disseminated out of Haiti since the early 1970s and reach a high prevalence (40–95%) in several countries throughout the Caribbean region including Haiti, Dominican Republic, Jamaica, Trinidad and Tobago, other Lesser Antilles, Suriname and French Guiana^19^,^20^,^21^. The non-pandemic B_CAR_ lineages did not remained restricted to the Caribbean region, but also reached most countries from Latin America^20^ and despite the overall low prevalence (<10%), established secondary outbreaks of small size in Argentina, Brazil, Mexico, Panama and Venezuela^20^,^21^. North America and Europe have maintained a high level of population movement with several Caribbean countries^22^,^23^,^24^, but the epidemiological relevance of non-pandemic B_CAR_ strains in those regions has not been explored.

The objective of this study was to estimate the prevalence of the HIV-1 B_PANDEMIC_ and B_CAR_ clades in North America and Europe and to reconstruct the spatiotemporal dynamics of dissemination of non-pandemic subtype B lineages detected in those regions. For this, we used a comprehensive dataset of HIV-1 subtype B *pol* sequences (*n* = 20,045) isolated from the US, Canada, and 26 European countries between 1982 and 2012. North American and European subtype B sequences were combined with reference sequences representative of the B_PANDEMIC_ and the B_CAR_ clades and then analyzed using Maximum Likelihood and Bayesian phylogeographic approaches.

## Materials and Methods

### HIV-1 subtype B *pol* sequence dataset

We downloaded all HIV-1 subtype B *pol* sequences from North America (*n* = 9,670) and Europe (*n* = 10,885) that covered the entire protease and partial reverse transcriptase (PR/RT) regions (nucleotides 2253–3260 relative to HXB2 clone) and were available at the Los Alamos HIV Database (http://www.hiv.lanl.gov) by July 2014. Only one sequence per subject was selected and those sequences containing frameshift mutations (1.4%) or with incorrect subtype B assignment (1.1%) according to the REGA HIV subtyping tool v.2^25^ were removed. This resulted in a final data set of 20,045 subtype B *pol* sequences isolated from North America (Canada and the US) and from Central (Austria, Germany, Poland, Slovakia and Switzerland), Northern (Denmark, Finland, Norway and Sweden), Southern (Italy, Portugal and Spain), Southeastern (Albania, Cyprus, Greece, Montenegro, Romania, Serbia and Slovenia) and Western (Belgium, Ireland, Luxembourg, Netherlands, France and the United Kingdom) Europe, between 1982 and 2012 (Table S1).

### Subtype B Clade assignment

HIV-1 subtype B *pol* sequences from North America and Europe were aligned with subtype B *pol* sequences representative of the B_PANDEMIC_ and the B_CAR_ clades described previously^19^,^21^, using the Clustal W program^26^. Codons associated with major antiretroviral drug resistance in PR (*n* = 12) and RT (*n* = 21) were excluded, resulting in a final alignment of 909 nucleotides. Sequences were initially classified as B_CAR_ or B_PANDEMIC_ by using an evolutionary placement algorithm (EPA)^27^ available in RAxML^28^ for the rapid assignment of query sequences to edges of a reference phylogenetic tree under a maximum-likelihood (ML) model. Sequences classified within the B_CAR_ clade were again combined with the B_PANDEMIC_ and the B_CAR_ reference sequences and subjected to new rounds of ML phylogenetic analysis with the PhyML program^29^ using an online web server^30^, for confirmation of clade assignment. Trees were inferred under the GTR+I+Γ nucleotide substitution model, selected using the jModeltest program^31^, and the SPR branch-swapping algorithm of heuristic tree search. The reliability of tree topology was estimated with the approximate likelihood-ratio test (*aLRT*)^32^ based on the Shimodaira-Hasegawa(SH)-like procedure. Clusters were classified as medium (SH-*aLRT* = 0.85–0.94) and well (SH-*aLRT* ≥ 0.95) supported, considering that the false positive error rate should not exceed 10% and 1% respectively^33^. Potential epidemiologic North American and European B_CAR_ clades were defined as those strongly supported monophyletic clusters exclusively composed by sequences from those geographic regions. The ML trees were visualized using the FigTree v1.4.0 program^34^.

### Analysis of the spatiotemporal dispersion pattern

The evolutionary rate, the age of the most recent common ancestor (*T*_MRCA_) and the spatial diffusion pattern of non-pandemic HIV-1 subtype B clades circulating in North America, Europe and the Caribbean were jointly estimated using the Bayesian Markov Chain Monte Carlo (MCMC) approach as implemented in BEAST v1.8^35^,^36^ with BEAGLE to improve run-time^37^. Analyses were performed using the GTR+I+Г_4_ nucleotide substitution model, a relaxed uncorrelated lognormal molecular clock model^38^, and a Bayesian Skyline coalescent tree prior^39^. The regression analysis of root-to-tip genetic distance against sampling time performed by using the program TempEst^40^, revealed that the HIV-1 subtype B dataset here compiled does not contain sufficient temporal signal for reliable time-scale estimation (X-intercept [*T*_MRCA_] = 1908). Thus, we specified a uniform prior distribution on the substitution rate (2.0–3.0 × 10^−3^ subst./site/year) that encompass mean values previously estimated for the subtype B *pol* gene^16^,^21^,^41^,^42^. Migration events throughout the phylogenetic history and the most relevant migration pathways were reconstructed using a reversible discrete phylogeography model and the Bayesian stochastic search variable selection (BSSVS) approach^43^, with a CTMC rate reference prior^44^. Discrete locations were assigned according to the sampling country of the sequences, except for European sequences that were grouped according to the sampling region (Central, Northern, Southern, Southeastern and Western, see Table S1) to reduce the complexity of the migration matrix. Three MCMC chains were run for 5 × 10^8^ generations and then combined using LogCombiner v1.8 while excluding the initial states (10–20%) of each run, needed to reach convergence. Convergence and uncertainty of parameter estimates were assessed by calculating the Effective Sample Size (ESS) and the 95% Highest Probability Density (HPD) values, respectively with Tracer v1.6^45^. The maximum clade credibility (MCC) tree was summarized with TreeAnnotator v1.8 and visualized with FigTree v1.4.0. Assuming that the posterior probability (*PP*) support closely reflected the probability for a split to be correct (under the true evolutionary model and correct priors distributions)^46^, clades were defined as moderate (*PP* = 0.85–0.94) or strongly (*PP* ≥ 0.95) supported. The cross-platform SPREAD application^47^ was used to summarize the migratory events and to identify statistically well-supported migration routes (those with Bayes factor [BF] > 3).

## Results

### Detection of HIV-1 BC_AR_ strains in North America and Europe

**Figure 1:**
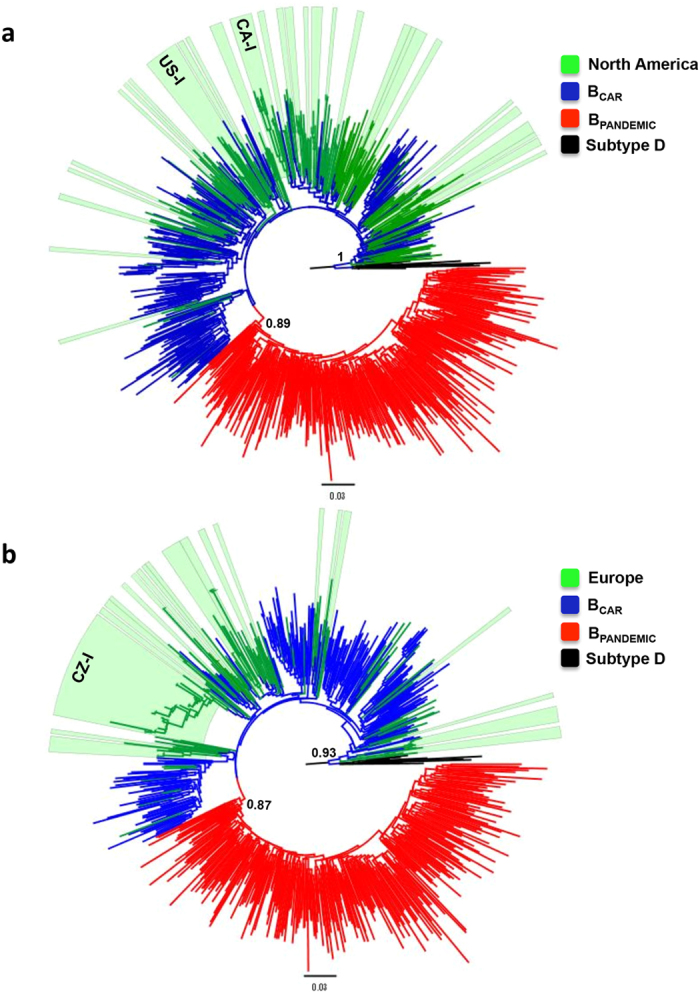
ML phylogenetic trees of HIV-1 B_CAR_ *pol* sequences identified in North America (**a**) and Europe (**b**). North American (*n* = 274) and European (*n* = 189) B_CAR_ sequences were combined with representative sequences of the B_PANDEMIC_ (US = 165, France = 135) and the B_CAR_ (Caribbean = 200) clades. Branches are colored according to the geographic origin/clade classification of each sequence as indicated at the legend (upper right). Colored boxes show the positions of strongly supported (SH-*aLRT* ≥ 0.95) North American and European B_CAR_ clades. Major (*n* ≥ 10) B_CAR_ clades detected in North America (US-I and CA-I) and Europe (CZ-I) are indicated. SH-a*LRT* supports for subtype B and B_PANDEMIC_ clades are shown. Trees were rooted using HIV-1 subtype D reference sequences. The branch lengths are drawn to scale with the bar at the bottom indicating nucleotide substitutions per site.

**Figure 2:**
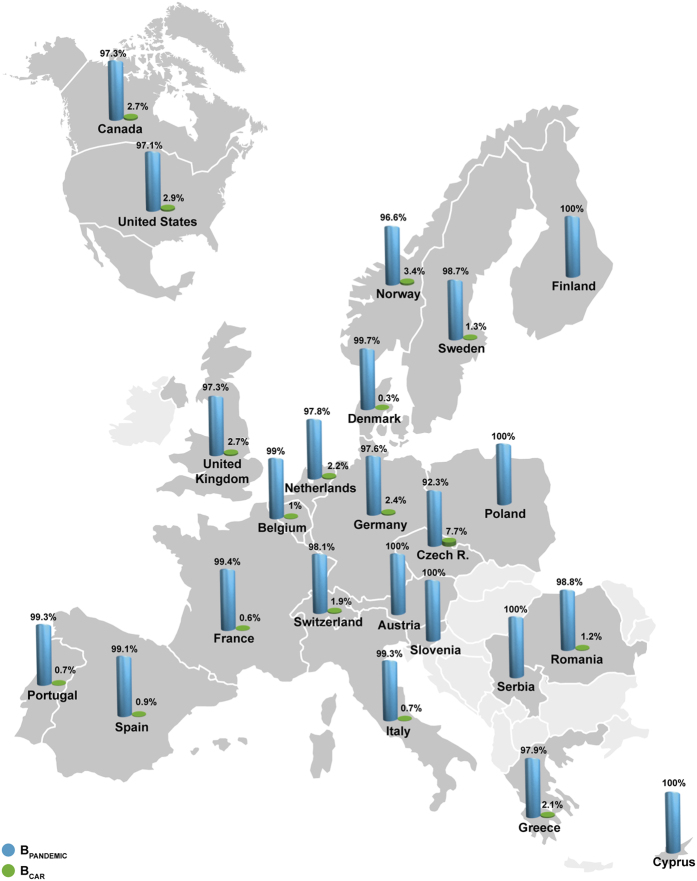
Estimated proportion of B_CAR_ and B_PANDEMIC_ clades among HIV-1 subtype B infected individuals from different North American and European countries. Maps were created with Adobe Illustrator CC from templates obtained from d-maps.com (North America: http://d-maps.com/pays.php?num_pay=119&lang=es; and Europe: http://d-maps.com/pays.php?num_pay=192&lang=es).

A total of 20,045 HIV-1 subtype B *pol* sequences from different countries from North America and Europe were combined with a set of B_PANDEMIC_ and B_CAR_ reference sequences^19^ and classified by using two sequential ML-based frameworks. This procedure classified a total of 274 (2.9%) and 189 (1.8%) HIV-1 subtype B *pol* sequences from North America and Europe within the B_CAR_ clades, respectively. The final ML phylogenetic trees clearly showed that the B_CAR_ sequences from North America and Europe were dispersed among the B_CAR_ reference sequences of Caribbean origin, while all B_PANDEMIC_ reference sequences branched in a medium supported (SH-*aLRT* = 0.85–0.90) clade nested within basal B_CAR_ reference sequences (Fig. 1). These analyses confirmed the circulation of B_CAR_ sequences in Canada, the US and 16 out of 26 European countries here analyzed (Fig. 2). Most European countries with detection of B_CAR_ strains encompass larger number of sequences (*n* > 250) than European countries with no evidence of circulation of B_CAR_ strains (*n* < 100) (Table S1). The B_CAR_ strains reached a low prevalence (<5%) among subtype B-infected individuals from all North American and European countries analyzed, with the only exception of the Czech Republic (CZ) where the B_CAR_ strains represent 7.7% of the sequences included (Fig. 2 and Table S1).

The ML phylogenetic analyses also revealed that many B_CAR_ strains from North America and Europe appear as sporadic lineages intermixed among Caribbean sequences, whereas other branched in strongly supported (aLRT ≥ 0.95) clades exclusively composed by sequences from those regions (Fig. 1). Most North American and European B_CAR_ clades were of small size (*n* < 10) and comprise sequences from a single country (country-specific clades) (Fig. S1 and Table S2). A few country-specific clades of large size (*n* = 11–48), however, were detected in Canada, the US, and the CZ (Fig. 1). We also detected a few international clades that encompass sequences from at least two different countries (Canada/US, Germany/Netherlands, CZ/Portugal/UK, Belgium/CZ/Norway, Germany/Netherlands/Switzerland/UK and CZ/Germany/Spain/Switzerland) (Fig. S1). In most countries analyzed the B_CAR_ sequences were mostly classified as sporadic lineages (57–67%), with exception of Canada, Germany and the CZ, where most B_CAR_ sequences (60–97%) were classified within country-specific/international clades (Table S2).

### Origin of HIV-1 B_CAR_ strains introduced in North America and Europe

HIV-1 B_CAR_ *pol* sequences with known sampling date from North America (*n *= 216) and Europe (*n *= 126) here identified, were next combined with B_CAR_ *pol* sequences from the most widely sampled (*n* > 10) Caribbean islands (*n* = 258)^19^,^21^ and with subtype D *pol* sequences (the closest relative to subtype B) from the Democratic Republic of Congo (the most probable epicenter of ancestral subtype B)^12^ (*n* = 10) that served as outgroup (Table S3). HIV-1 sequences were classified into 10 discrete geographic locations and subjected to Bayesian phylogeographic analyses. When all B_CAR_ sequences were combined in a single dataset, the MCMC chains fail to converge and many parameters showed low (<100) ESS values despite very long runs (1 × 10^9^ generations). Furthermore, the most probable ancestral root location for the subtype B epidemic was traced to the US (posterior state probability [*PSP*] > 0.90), which is inconsistent with the seminal work of Gilbert and colleagues (2007) that clearly traced the origin of American subtype B epidemic to Haiti. Sampling bias can confuse the accurate estimation of the spatial root of an epidemic^48^ and our convenience sampling was clearly biased to the US (*n* = 164) (Table S3). Moreover, the B_CAR_ US sequences were widely scattered among B_CAR_ sequences of Hispaniola (probably due to many independent viral introductions into the US), so the B_CAR_ genetic diversity in both locations was comparable and the simple overrepresentation of the US may bias the ancestral root location to this country.

**Figure 3:**
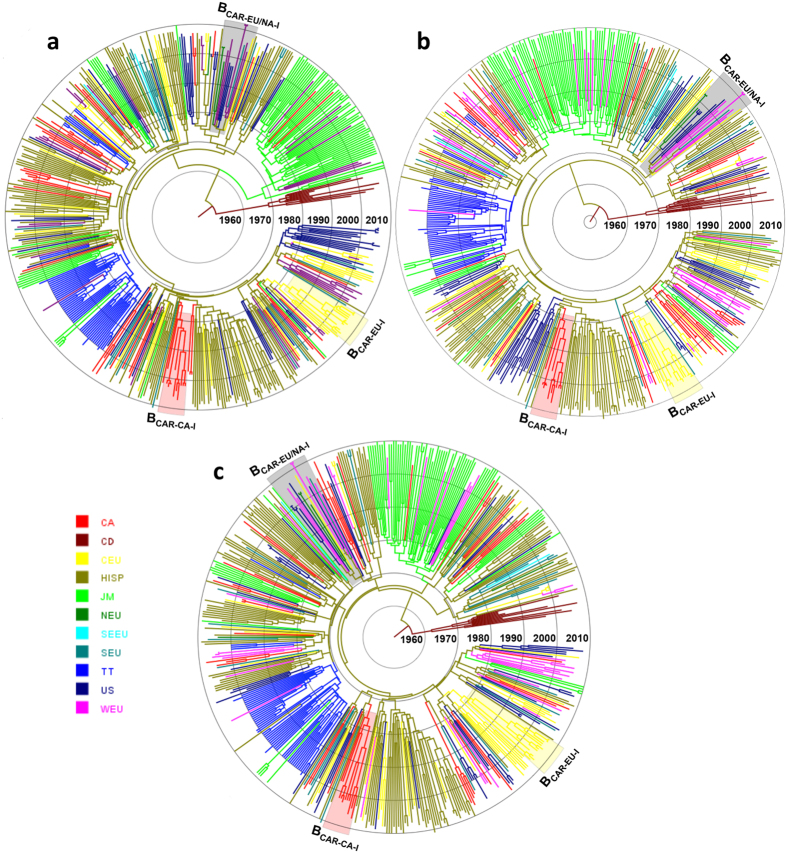
Time-scaled Bayesian MCMC tree of HIV-1 B_CAR_ *pol* sequences from North America and Europe. North American (*n *= 216) and European (*n *= 126) B_CAR_ sequences with known sampling date were combined with B_CAR_ sequences from the Caribbean (*n* = 258), B_PANDEMIC_ sequences (*n* = 50) the US and France and subtype D sequences (*n* = 10) from the DRC. Branches of trees reconstructed from subsets 1 (**a**), 2 (**b**), and 3 (**c**) are colored according to the most probable location state (see Table S3) of their descendent nodes as indicated in the legend (bottom left). Colored boxes indicate the positions of major (*n* ≥ 10) B_CAR_ clades detected outside the Caribbean. Branch lengths are depicted in units of time (years). The trees were automatically rooted under the assumption of a relaxed molecular clock. CA: Canada, CD: Democratic Republic of Congo, CEU: Central Europe, HISP: Hispaniola, JM: Jamaica, NEU: Northern Europe, SEEU: Southeastern Europe, SEU: Southern Europe, TT: Trinidad and Tobago, US: United States of America, WEU: Western Europe.

**Figure 4:**
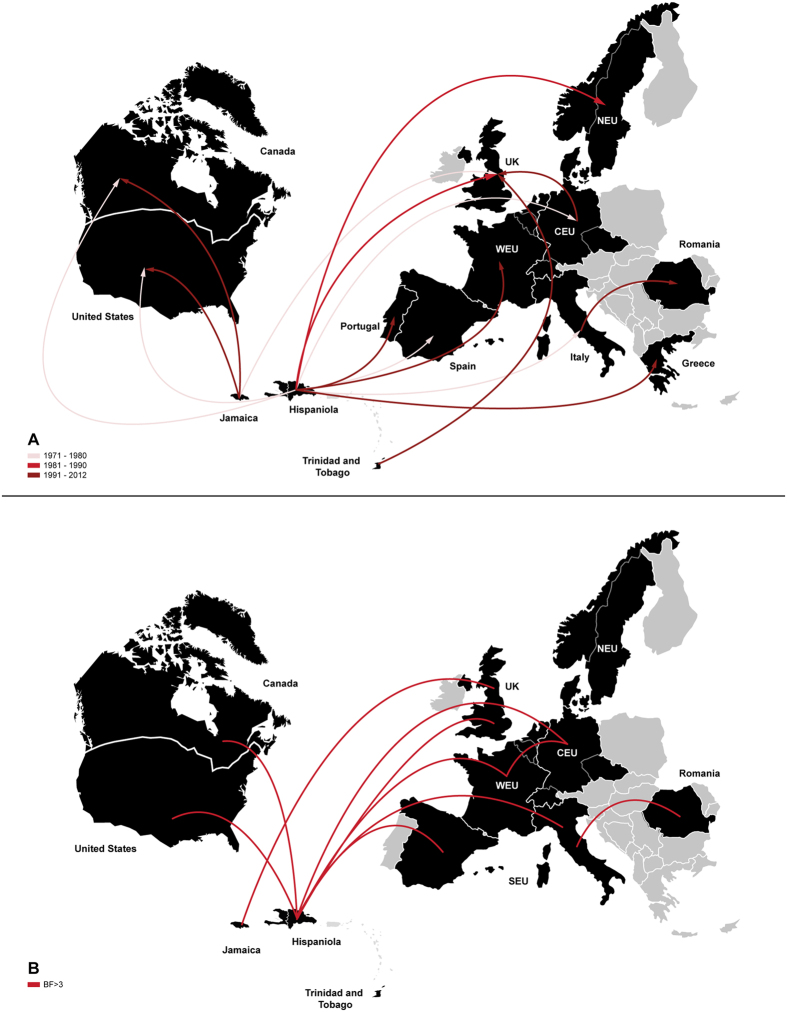
Spatiotemporal dynamics of dissemination of HIV-1 B_CAR_ clades from the Caribbean to North America and Europe. (**a**) Lines between locations represent branches in the Bayesian MCC trees along which viral migration events occurred. The line’s color informs the estimated time interval of the viral migrations as indicated in the legend (bottom left). Only the earliest transitions between each location pair were represented. (**b**) Most significant (Bayes factor rates > 3) epidemiological links of the dissemination process of B_CAR_ clades. CEU: Central Europe, NEU: Northern Europe, WEU: Western Europe. UK: United Kingdom. Maps were created with Adobe Illustrator CC from templates obtained from d-maps.com (Caribbean: http://d-maps.com/pays.php?num_pay=118&lang=es; North America: http://d-maps.com/pays.php?num_pay=119&lang=es; and Europe: http://d-maps.com/pays.php?num_pay=192&lang=es).

In order to reduce the impact of sampling bias on the accuracy of phylogeographic reconstructions, sequences from the US were subdivided into three subsets (*n* = 54–55) at random, except for those sequences belonging to the small country-specific clades (*n* ≤ 5) that were placed in the same subset (Table S4) in order to recover their *T*_MRCA_. Each subset was then combined with sequences from the other locations and independently analyzed. The overall pattern of spatial and temporal dissemination of B_CAR_ strains reconstructed from all subsets was roughly similar (Fig. 3). All three analyses pointed to the island of Hispaniola as the most probable root location of the HIV-1 subtype B ancestor (*PSP* = 0.76–0.99), and traced the median T_MRCA_ of subtype B at around the middle 1960s (Table 1), fully consistent with previous estimates^12^,^49^,^50^. Phylogeographic reconstructions also suggest that Hispaniola was the major hub of international dissemination of B_CAR_ strains, sending viruses to Canada, the US and all European regions at multiple times from the middle 1970 s onwards (Fig. 4A). Additional viral migrations from Jamaica to Canada/US/UK and from Trinidad and Tobago to the UK were also detected (Fig. 4A). The BF tests for significant nonzero rates support epidemiological linkage between: Hispaniola/Canada, Hispaniola/US, Hispaniola/Central Europe, Hispaniola/Southern Europe, Hispaniola/Western Europe and Jamaica/Western Europe (UK) (Fig. 4B).

### Onward transmission of HIV-1 B_CAR_ strains in North America and Europe

The Bayesian analysis confirms that some B_CAR_ strains seeded secondary outbreaks in North America and Europe (Fig. 3). The overall distribution of B_CAR_ sequences across sporadic lineages and country-specific/international clades was comparable to that inferred from the ML trees (Table S2). Nearly all highly supported North American and European clades previously identified also displayed a very high support in Bayesian trees (*PP* = 1) (Table S5). Bayesian analyses also recovered three medium/highly supported (*PP* = 0.90–0.99) European B_CAR_ lineages (B_CAR-EU-I,_ B_CAR-EU-V_ and B_CAR-EU-VI_) that were observed as low/medium supported (a*LRT* = 0.82–0.89) clades in the ML trees (Table S5). Finally, the combination of North American and European sequences in the same dataset allowed the identification of three medium/highly supported (*PP* = 0.88-1) intercontinental B_CAR_ clades (Fig. 3 and Table S5). The major intercontinental clade (B_CAR-NA/EU-I_) comprises sequences from the US and six European countries, the second one (B_CAR-NA/EU-II_) comprises sequences from the US, Canada, Belgium, the CZ and Norway, and the last one (B_CAR-NA/EU-III_) comprises sequences from the US and Spain.

A great proportion (40%) of North American and European B_CAR_ clades probably arose between the late 1970 s and the late 1980 s, others (46%) arose during the 1990 s, and a minor fraction (14%) arose during the 2000 s (Table 1). Most (82%) North American and European B_CAR_ clades seem to have remained confined to one single country. A few European B_CAR_ clades were disseminated across neighboring countries such the CZ, Germany and Switzerland (B_CAR-EU-I_ and B_CAR-EU-II_) or Germany and Netherlands (B_CAR-EU-VI_) and others were spread over longer distances, like those disseminated from Central Europe to UK (B_CAR-NA/EU-III_ and B_CAR-NA/EU-IV_), and from Italy to Romania (B_CAR-EU-V_) (Fig. 4A). The most notable examples of long-distance dissemination, however, were the three intercontinental B_CAR_ clades that probably arose between the early 1970 s and the early 1980 s and were spread between North America and Europe.

**Figure 5:**
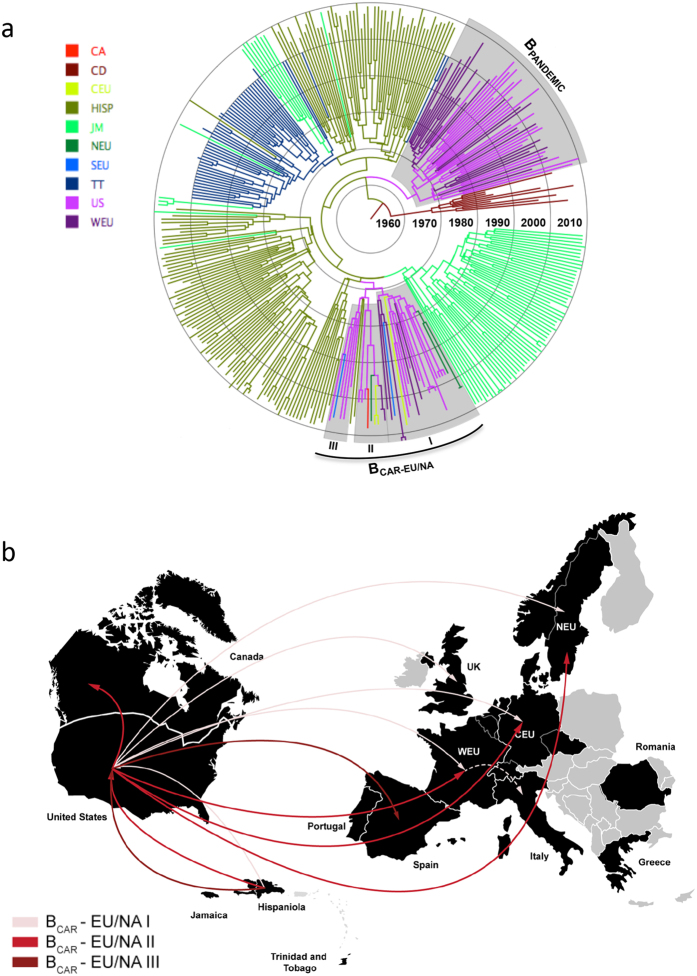
Spatiotemporal dynamics of dissemination of HIV-1 B_CAR_ clades from North America to Europe. (**a**) Time-scaled Bayesian MCMC tree of HIV-1 B_CAR_ *pol* sequences from intercontinental B_CAR_ clades (B_CAR-NA/EU-I_, B_CAR-NA/EU-II_ and B_CAR-NA/EU-III_) combined with B_CAR_ sequences from the Caribbean (*n *= 258), B_PANDEMIC_ sequences from the US and France (*n *= 50) and subtype D sequences (*n* = 10) from the DRC. Branches are colored according to the most probable location state (see Table S3) of their descendent nodes as indicated in the legend (upper left). Colored boxes indicate the positions of intercontinental B_CAR_ clades and the B_PANDEMIC_ clade. Branch lengths are depicted in units of time (years). The tree was automatically rooted under the assumption of a relaxed molecular clock. See the legend of Fig. 3 for location code. (**b**) Lines between locations represent branches in the Bayesian MCC tree along which viral migration events occurred. Dispersion pathways of different intercontinental B_CAR_ clades are represented with different colors (see the legend at bottom left). Epidemiological links supported by Bayes factor rates >3 and <3 are displayed with continuous and discontinuous lines, respectively. See the legend of Fig. 4 for location code. Maps were created with Adobe Illustrator CC from templates obtained from d-maps.com (Caribbean: http://d-maps.com/pays.php?num_pay=118&lang=es; North America: http://d-maps.com/pays.php?num_pay=119&lang=es; and Europe: http://d-maps.com/pays.php?num_pay=192&lang=es).

In order to gain a better understanding of their dissemination dynamics, the three intercontinental B_CAR_ clades were combined in a single dataset with B_CAR_ strains from the Caribbean (*n* = 258), a representative sub-set of B_PANDEMIC_ sequences from the US and France (*n* = 50) and subtype D sequences from the DRC (*n* = 10). The Bayesian analysis clearly reconstructed long distance migrations of clades B_CAR-NA/EU-I_, B_CAR-NA/EU-II_ and B_CAR-NA/EU-III_ from the US to Europe at multiple times, supported by high BF rates (Fig. 5). The T_MRCA_ of the B_PANDEMIC_ clade was estimated at 1969 (1965–1973), consistent with a previous study^12^, of the B_CAR-NA/EU-I_ clade at 1972 (1968–1976), of the B_CAR-NA/EU-II_ clade at 1977 (1972–1982), and of the of the B_CAR-NA/EU-III_ clade at 1978 (1973–1982). This phylogeographic analysis further suggests that clades B_CAR-NA/EU-I_, B_CAR-NA/EU-II_ and B_CAR-NA/EU-III_ may have evolved from a single ancestor traced to the US at 1970 (1966–1974), but this result should be interpreted with caution because the support for that large intercontinental B_CAR_ clade was low (*PP* = 0.59).

## Discussion

This study confirms that the HIV-1 subtype B epidemic in North America and Europe is mostly driven by the dissemination of the B_PANDEMIC_ lineage, but also demonstrates several independent introductions of non-pandemic B_CAR_ strains of Caribbean origin into those regions. The dissemination of B_CAR_ strains from the Caribbean into North America and Europe should be expected considering the high human mobility between those regions. The discovery of the Americas in 15^th^ century was the starting point for several European countries to create colonies in the Caribbean region that persisted for several centuries and many of them continue to have government ties with European countries (such as the UK, France and the Netherlands) and the US at the 21^st^ century. The linguistic and socioeconomic links created during the colonial period certainly facilitated: (1) a large flow of labor migrants from Caribbean countries towards North America and Europe, particularly since 1970^22^,^23^,^24^, and (2) a sharp increase in the number of visitors mainly from the US, Germany, the UK, France and Canada towards the Caribbean, particularly since the 1960 s when regular international airplane flights made vacations to the Caribbean more affordable^23^,^51^.

Our results indicate that the island of Hispaniola was probably the major source of B_CAR_ lineages disseminated into those regions. Jamaica can be viewed as a secondary hub sending B_CAR_ strains to the US, Canada and the UK, whereas Trinidad and Tobago seems to have played a minor role in long-distance dispersion of B_CAR_ strains. It could be argued that these estimates were biased by the sampling scheme used here since, after down-sampling the US, most B_CAR_ sequences were from Hispaniola. If we considered the relative contribution of each location to the total number of B_CAR_-infected individuals, however, our estimates are quite robust to sampling bias since all locations were overrepresented with the only exception of Hispaniola. This Caribbean island hosts about 70% of the total number of B_CAR_-infected individuals, but only comprises 27% of B_CAR_ sequences in our subsets. Our convenience sampling, however, probably failed to recover some important dispersal routes between Caribbean countries with high prevalence of B_CAR_ lineages and strong connection with North America/Europe, like many Lesser Antilles islands, Guyana, French Guyana and Suriname^19^,^20^, that were not represented in our subsets.

Some viral migration routes here recovered are fully consistent with the notion that postcolonial ties are an important driving force in the international dissemination of B_CAR_ strains. The spread of these strains out of Jamaica, for example, was traced to those countries (the US, the UK and Canada) where most Jamaicans immigrants reside and from where most tourists visiting Jamaica originate^22^,^23^,^24^. Other factors apart from the historical, linguistic and socio-economic links with the Caribbean region, however, appear to be needed to explain the overall prevalence and distribution of B_CAR_ strains across different European countries. We observed a very low prevalence (<1%) of B_CAR_ strains among subtype B-infected individuals from some countries (France and Spain) that host large numbers of Caribbean immigrants and contribute many tourists to the Caribbean region, and a relative high prevalence of B_CAR_ strains (2–8%) in other countries (the CZ, Greece, Luxembourg and Norway) located outside the main migration corridors from/to the Caribbean^22^,^23^,^24^.

Stochastic events may have influenced the B_CAR_ circulation in some countries like the CZ, where most (73%) B_CAR_ sequences branched in a country-specific sub-clade that was nested among basal B_CAR_ sequences from Germany and Switzerland. This supports that the high prevalence of B_CAR_ sequences observed in the CZ probably resulted from the local expansion of a single founder strain introduced from a neighboring European country. Such a founder B_CAR_ strain probably gained access to some highly interconnected network of IDU or MSM from the CZ, although we have no epidemiological information about the B_CAR_-infected patients to test this hypothesis. Other country-specific B_CAR_ clades were also detected in the US, Canada, Germany, Italy, Spain and the UK, supporting local dissemination of non-pandemic subtype B lineages in a number of North American and European countries. In addition, we also detected short-distance spreading of B_CAR_ lineages between neighboring European countries from Central and Western Europe, and long-distance dissemination between the US and Europe.

While stochastic founder events may help to explain the dissemination of B_CAR_ strains in countries that are not part of the main migration corridors with the Caribbean, cultural factors could be a major obstacle for the onward transmission of B_CAR_ strains primarily introduced into countries strongly connected to the Caribbean. Migrant travelers might form a bridge population for HIV transmission between their country of origin and their country of residence. It has been estimated, for example, that about 6% of the Surinamese and Antillean migrant population act as a potential bridge population for HIV transmission in The Netherlands^52^. Molecular epidemiological data shows, however, that HIV transmission occurs mostly within migrant communities, whereas transmission between Surinamese, Antillean, and Dutch individuals living in The Netherlands are rare^53^. Consequently, the B_CAR_ strains potentially introduced into The Netherlands from Suriname and the Netherlands Antilles should not be expected to fuel large outbreaks among indigenous Dutch population.

Although the time-scale here reconstructed was largely determined by the informative prior distribution specified on substitution rate, the T_MRCA_ estimated for subtype B/D, subtype B and the B_PANDEMIC_ clades were fully consistent with those obtained in previous studies^12^,^49^,^50^, thus indicating that our informative prior produced reliable time-scale estimates. According to our estimations, the *T*_MRCA_ of most (77%) international B_CAR_ clades detected in North America and Europe was traced to between the early 1970 s and the early 1980 s, whereas the onset date of a significant fraction (40%) of country-specific B_CAR_ clades detected in those regions was traced to between the late 1970 s and the late 1980 s. This demonstrates that despite their low prevalence, the B_CAR_ strains were introduced and circulate in North America and Europe since the early stages of the AIDS epidemic. In sharp contrast to the B_PANDEMIC_ clade, however, all B_CAR_ introductions in North America and Europe seem to have resulted in dead-end infections or in outbreaks of small size, as previously demonstrated for most Latin American countries^20^.

One interesting question is why B_CAR_ strains introduced into North America and Europe fail to establish large secondary outbreaks. One hypothesis is that the B_PANDEMIC_ clade displays a higher transmissibility than B_CAR_ clades, but some evidences argue against it. First, analysis of partial genome regions revealed a paucity of amino acid substitutions mapping onto the branch leading to the B_PANDEMIC_ ancestor^12^, suggesting that this clade probably possessed no selective advantage over B_CAR_ strains. Second, in several Caribbean countries where both pandemic and non-pandemic lineages co-circulate, the B_CAR_ clades reached a much higher prevalence than the B_PANDEMIC_ one^19^,^20^. Another hypothesis is that the epidemic outcome of different subtype B strains was mostly shaped by ecological factors, like the chance of colonization of different populations. It is possible that the B_PANDEMIC_ ancestor was the only subtype B Caribbean strain to gain access to the globally interconnected populations of MSM and IDU from the US, Europe and Latin America at the early stages of the epidemic^15^, thus resulting in a much more efficient dissemination at both local and global level.

In conclusion, this study demonstrates that several non-pandemic HIV-1 B_CAR_ strains have been introduced from the Caribbean into North America and Europe since the early 1970 s onwards. Some B_CAR_ strains were spread locally in the US, Canada and several European countries. We further detected short-distance spreading of B_CAR_ lineages between neighboring European countries and long-distance disseminations between the US and Europe. Despite their early and frequent introduction, the B_CAR_ strains only comprise a very low fraction of all HIV-1 subtype B infections from North America (3%) and Europe (1%). The epidemiological characterization of the transmission networks that sustain the dissemination of the B_CAR_ clades in North America and Europe will be of paramount importance to determine why no large B_CAR_ outbreaks have been established in those regions.

## Additional Information

**How to cite this article**: Cabello, M. *et al*. Multiple introductions and onward transmission of non-pandemic HIV-1 subtype B strains in North America and Europe. *Sci. Rep.* **6**, 33971; doi: 10.1038/srep33971 (2016).

## Supplementary Material

Supplementary Information

## Figures and Tables

**Table 1:** Bayesian estimates of the T_MRCA_ of HIV-1 B_CAR_ clades from North America and Europe.

Clade	Countries	Subset 1 T_MRCA_	Subset 2 T_MRCA_	Subset 3 T_MRCA_
Subtypes B/D	‒	1956 (1947–1964)	1956 (1948–1964)	1957 (1948–1964)
Subtype B	‒	1967 (1963–1971)	1967 (1964–1970)	1968 (1964–1971)
B_CAR-NA/EU-I_	DE/FR/IT/LU/SE/US/UK	1974 (1971–1977)	1974 (1970–1976)	1974 (1971–1977)
B_CAR-NA/EU-II_	BE/CA/CZ/NO/US	1978 (1974–1982)	‒	‒
B_CAR-NA/EU-III_	US/ES	1980 (1976–1983)	‒	‒
B_CAR-EU-I_	CH/CZ/DE	1981 (1977–1985)	1981 (1977–1985)	1981 (1977–1986)
B_CAR-EU-II_	CH/CZ/DE	1979 (1976–1983)	1979 (1975–1983)	1980 (1976–1983)
B_CAR-EU-III_	CH/DE/UK	1992 (1986–1996)	1992 (1987–1996)	1992 (1987–1996)
B_CAR-EU-IV_	CZ/UK	1997 (1994–2000)	1997 (1994–2000)	1997 (1994–2000)
B_CAR-EU-V_	IT/RO	1980 (1976–1983)	1979 (1976–1983)	1980 (1976–1983)
B_CAR-EU-VI_	DE/NL	1982 (1977–1987)	1983 (1978–1989)	1982 (1977–1987)
B_CAR-CZ-I_	CZ	1996 (1992–1999)	1996 (1992–1999)	1996 (1992–1999)
B_CAR-US-II_	US	‒	‒	1994 (1991–1997)
B_CAR-US-III_	US	‒	‒	1987 (1982–1994)
B_CAR-US-IV_	US	‒	1989 (1983–1993)	‒
B_CAR-US-V_	US	2005 (2004–2006)	‒	‒
B_CAR-US-VI_	US	‒	‒	1994 (1988–2000)
B_CAR-US-VII_	US	‒	1994 (1991–1996)	‒
B_CAR-US-VIII_	US	‒	‒	1985 (1982–1989)
B_CAR-US-IX_	US	‒	1991 (1985–1996)	‒
B_CAR-US-X_	US	‒	‒	1996 (1995–1997)
B_CAR-US-XII_	US	‒	1995 (1991–1999)	‒
B_CAR-US-XIII_	US	‒	‒	1995 (1994–1997)
B_CAR-US-XIV_	US	2004 (2002–2005)	‒	‒
B_CAR-US-XV_	US	‒	‒	2005 (2002–2006)
B_CAR-US-XIX_	US	‒	‒	1982 (1978–1987)
B_CAR-CA-I_	CA	1979 (1975–1982)	1978 (1975–1982)	1979 (1975–1983)
B_CAR-CA-II_	CA	1996 (1988–2002)	1996 (1988–2002)	1996 (1988–2002)
B_CAR-CA-III_	CA	1983 (1979–1987)	1983 (1979–1987)	1984 (1979–1988)
B_CAR-CA-IV_	CA	1998 (1994–2001)	1998 (1994–2001)	1998 (1994–2001)
B_CAR-CA-V_	CA	1987 (1981–1993)	1987 (1981–1992)	1987 (1982–1993)
B_CAR-CA-VI_	CA	1998 (1993–2002)	1998 (1993–2002)	1998 (1993–2002)
B_CAR-CA-VII_	CA	1998 (1997–1998)	1998 (1996–1998)	1998 (1997–1998)
B_CAR-DE-II_	DE	1988 (1982–1993)	1987(1982–1994)	1988 (1982–1993)
B_CAR-DE-III_	DE	1987 (1981–1994)	1987 (1980–1993)	1988 (1982–1994)
B_CAR-ES-I_	ES	2006 (2004–2008)	2006 (2004–2008)	2006 (2004–2008)
B_CAR-IT-II_	IT	1989 (1983–1993)	2006 (2004–2008)	2006 (2004–2008)
B_CAR-UK-I_	UK	1993 (1990–1996)	1993 (1990–1996)	1994 (1990–1996)
B_CAR-UK-II_	UK	2004 (2002–2004)	2004 (2002–2004)	2004 (2002–2004)
B_CAR-UK-III_	UK	1994 (1988–1999)	1994 (1988–1998)	1994 (1988–1999)
