# Supplementary material for: Multiple introductions and onward transmission of non-pandemic HIV-1 subtype B strains in North America and Europe
Source: Sci Rep. 2016 Sep 22;6:33971. doi: 10.1038/srep33971 (PMC5032033; doi:10.1038/srep33971)
Supplement: Supplementary Information [file srep33971-s1.pdf]

Supplementary Information

Title: Multiple introductions and onward transmission of non-pandemic HIV-1 subtype B strains in North America and Europe.

Authors: Marina Cabello, Hector Romero and Gonzalo Bello

Table S1. Clade assignment of HIV-1 subtype B subtype *pol* sequences from North America and Europe.

Region	Country	*N*	$B_{PANDEMIC}$	B_{CAR}
North America	Canada	2,769	2,693 (97.3%)	76 (2.7%)
	US	6,771	6,573 (97.1%)	198 (2.9%)
	**Total**	**9,540**	**9,266 (97.1%)**	**274 (2.9%)**
Central Europe	Austria	79	79 (100%)	0
	Czech Republic	861	795 (92.3%)	66 (7.7%)
	Germany	1,045	1,020 (97.6%)	25 (2.4%)
	Poland	91	91 (100%)	0
	Slovakia	15	15 (100%)	0
	Switzerland	484	475 (98.1%)	9 (1.9%)
	**Total**	**2,575**	**2,475 (96.1%)**	**100 (3.9%)**
Northern Europe	Denmark	398	397 (99.7%)	1 (0.3%)
	Finland	41	41 (100%)	0
	Norway	58	56 (96.6%)	2 (3.4%)
	Sweden	309	305 (98.7%)	4 (1.3%)
	**Total**	**806**	**799 (99.1%)**	**7 (0.9%)**
Southern Europe	Italy	2,429	2,412 (99.3%)	17 (0.7%)
	Portugal	289	287 (99.3%)	2 (0.7%)
	Spain	1,556	1,542 (99.1%)	14 (0.9%)
	**Total**	**4,274**	**4,241 (99.2)**	**33 (0.8%)**
Southeastern Europe	Albania	23	23 (100%)	0
	Cyprus	90	90 (100%)	0
	Greece	48	47 (97.9%)	1 (2.1%)
	Montenegro	27	27 (100%)	0
	Romania	84	83 (98.8%)	1 (1.2%)
	Serbia	169	169 (100%)	0
	Slovenia	97	97 (100%)	0
	**Total**	**538**	**536 (99.6%)**	**2 (0.4%)**
Western Europe	Belgium	294	291 (99.0%)	3 (1%)
	Ireland	6	6 (100%)	0
	Luxembourg	26	25 (96.1%)	1 (3.9%)
	Netherlands	414	405 (97.8%)	9 (2.2%)
	France	479	476 (99.4%)	3 (0.6%)
	United Kingdom	1,093	1,063 (97.3%)	30 (2.7%)
	**Total**	**2,312**	**2,266 (98.0%)**	**46 (2.0%)**

Table S2. Number of HIV-1 B_{CAR} *pol* sequences from North America and Europe that were classified as sporadic lineages (SL) or within local (country-specific) clusters (LC) or international clusters (IC), according to the ML and Bayesian phylogenetic analyses.

Region	Country	Maximum Likelihood (SH-*aLRT* ≥ 0.95)			Bayesian (*PP* ≥ 0.85)		
		SL	LC	IC	SL	LC	IC
North America	Canada	30 (39%)	44 (58%)	2 (3%)	19 (37%)	32 (61%)	1 (2%)
	US	133 (67%)	63 (32%)	2 (1%)	96 (59%)	46 (28%)	22 (13%)
	**Total**	**163 (59%)**	**107 (39%)**	**4 (2%)**	**115 (53%)**	**78 (36%)**	**23 (11%)**
Central Europe	Czech Republic	2 (3%)	52 (79%)	12 (18%)	-	2 (13%)	14 (87%)
	Germany	10 (40%)	9 (36%)	6 (24%)	10 (40%)	8 (32%)	7 (28%)
	Switzerland	7 (78%)	-	2 (22%)	7 (78%)	-	2 (22%)
	**Total**	**19 (20%)**	**61 (60%)**	**20 (20%)**	**17 (34%)**	**10 (20%)**	**23 (46%)**
Northern Europe	Denmark	1 (100%)	-	-	1 (100%)	-	-
	Norway	1 (50%)	-	1 (50%)	1 (50%)	-	1 (50%)
	Sweden	2 (50%)	2 (50%)	-	1 (25%)	-	3 (75%)
	**Total**	**4 (57%)**	**2 (29%)**	**1 (14%)**	**3 (43%)**	**-**	**4 (57%)**
Southern Europe	Italy	9 (53%)	8 (47%)	-	5 (42%)	2 (16%)	5 (42%)
	Portugal	1 (50%)	-	1 (50%)	1 (100%)	-	-
	Spain	9 (64%)	4 (29%)	1 (7%)	10 (77%)	2 (15%)	1 (8%)
	**Total**	**19 (58%)**	**12 (36%)**	**2 (6%)**	**16 (62%)**	**4 (15%)**	**6 (23%)**
Southeastern Europe	Greece	1 (100%)	-	-	1 (100%)	-	-
	Romania	1 (100%)	-	-	-	-	1 (100%)
	**Total**	**2 (100%)**	**-**	**-**	**1 (50%)**	**-**	**1 (50%)**
Western Europe	Belgium	1 (33%)	-	2 (66%)	1 (33%)	-	2 (66%)
	Luxembourg	1 (100%)	-	-	-	-	1 (100%)
	Netherlands	5 (56%)	2 (22%)	2 (22%)	3 (60%)	-	2 (40%)
	France	1 (33%)	2 (66%)	-	-	-	2 (100%)
	United Kingdom	20 (67%)	7 (23%)	3 (10%)	17 (57%)	7 (23%)	6 (20%)
	**Total**	**28 (61%)**	**11 (24%)**	**7 (15%)**	**21 (51%)**	**7 (17%)**	**13 (32%)**

Table S3. HIV-1 B_{CAR} *pol* sequences from the Caribbean, North America and Europe used for Bayesian phylogeographic analysis. The asterisk (*) indicates B_{CAR} sequences identified in previous studies[19,21].

Region	Country	Location	*N*	Sampling interval
Caribbean*	Dominican Republic	HIS	123	2003-2011
	Haiti		12	2004-2005
	Jamaica	JM	73	2005-2010
	Trinidad and Tobago	TT	50	2000-2003
	**Total**	-	**258**	**2000-2011**
North America	US	US	164	1983-2009
	Canada	CA	52	1996-2009
	**Total**	-	**216**	**1983-2009**
Central Europe	Czech Republic	CEU	16	2000-2007
	Germany		25	1998-2008
	Switzerland		9	1996-2007
	**Total**		**50**	**1996-2008**
Northern Europe	Denmark	NEU	1	2000
	Norway		2	2004-2006
	Sweden		4	1998-2004
	**Total**		**7**	**1998-2006**
Southern Europe	Italy	SEU	12	1997-2006
	Portugal		1	2005
	Spain		13	2002-2011
	**Total**		**26**	**1997-2011**
Southeastern Europe	Greece	SEEU	1	2004
	Romania		1	2004
	**Total**		**2**	**2004**
Western Europe	Belgium	WEU	3	2003-2006
	Luxembourg		1	2006
	Netherlands		5	2003
	France		2	2012
	United Kingdom		30	1997-2007
	**Total**		**41**	**1997-2012**

The following HIV-1 subtype D *pol* sequences from the DRC were also included as outgroup: A34828, A07108, U88822, M22639, AJ287019, AF357631, AM041047, AM041035, KC340636, FR666667.

Table S4. Distribution of HIV-1 B_{CAR} *pol* sequences from the US across subsets.

Clade	Number of sequences	Number of sequences with sampling date	Subset 1	Subset 2	Subset 3
Non-clustered sequences	133	109	43	37	29
US-I	17	14	3	6	5
US-II	5	4	-	-	4
US-III	4	4	-	-	4
US-IV	4	4	-	4	-
US-V	3	3	3	-	-
US-VI	3	3	-	-	3
US-VII	3	3	-	3	-
US-VIII	3	3	-	-	3
US-IX	3	2	-	2	-
US-X	2	2	-	-	2
US-XI	2	2	2	-	-
US-XII	2	2	-	2	-
US-XIII	2	2	-	-	2
US-XIV	2	2	2	-	-
US-XV	2	2	-	-	2
US-XVI	2	1	-	1	-
US-XVII	2	-	-	-	-
US-XVIII	2	-	-	-	-
US/CA-I	1	1	1	-	-
US/CA-II	1	-	-	-	-
**Total**	**198**	**164**	**54**	**55**	**55**

Table S5. North American and European HIV-1 B_{CAR} *pol* clades with medium or high support detected in Bayesian and ML analyses.

**Bayesian analyses**		**ML analyses**	
**Country-specific clades**	***PP***	**Country-specific clades**	**SH-a*LRT***
$B_{CAR-US-II}$	1	US-II	1
$B_{CAR-US-III}$	1	US-III	0.98
$B_{CAR-US-IV}$	1	US-IV	0.99
$B_{CAR-US-V}$	1	US-V	1
$B_{CAR-US-VI}$	1	US-VI	1
$B_{CAR-US-VII}$	1	US-VII	1
$B_{CAR-US-VIII}$	1	US-VIII	1
$B_{CAR-US-IX}$	1	US-IX	1
$B_{CAR-US-X}$	1	US-X	1
$B_{CAR-US-XII}$	1	US-XII	0.99
$B_{CAR-US-XIII}$	1	US-XIII	1
$B_{CAR-US-XIV}$	1	US-XIV	1
$B_{CAR-US-XV}$	1	US-XV	1
$B_{CAR-US-XIX}$	0.97	US	0.92
$B_{CAR-CA-I}$	1	CA-I	0.95
$B_{CAR-CA-II}$	1	CA-II	0.99
$B_{CAR-CA-III}$	1	CA-III	0.99
$B_{CAR-CA-IV}$	1	CA-IV	1
$B_{CAR-CA-V}$	1	CA-V	0.96
$B_{CAR-CA-VI}$	1	CA-VI	0.96
$B_{CAR-CA-VII}$	1	CA-VII	1
$B_{CAR-DE-II}$	1	DE-II	0.98
$B_{CAR-DE-III}$	1	DE-III	0.99
$B_{CAR-ES-I}$	1	ES-I	1
$B_{CAR-IT-II}$	1	IT-II	1
$B_{CAR-UK-I}$	1	UK-I	0.99
$B_{CAR-UK-II}$	1	UK-II	1
$B_{CAR-UK-III}$	1	UK-III	0.99
**International clades**	***PP***	**International clades**	**SH-a*LRT***
$B_{CAR-NA/EU-I}$	0.88	US-I + FR-I + SE-I + DE + IT + LU + UK	-
$B_{CAR-NA/EU-II}$	0.99	US-XI + US/CA-I + BE/CZ/NO	-
$B_{CAR-NA/EU-III}$	1	US + ES	-
$B_{CAR-EU-I}$	0.90	CZ-I + DE-I + CH	0.86
$B_{CAR-EU-II}$	0.66	CH/CZ/DE/ES	0.95
$B_{CAR-EU-III}$	1	CH/DE/NL/UK	1
$B_{CAR-EU-IV}$	1	CZ/PT/UK	1
$B_{CAR-EU-V}$	0.99	IT-I + IT + RO	0.89
$B_{CAR-EU-VI}$	0.98	NL-I + DE	0.82

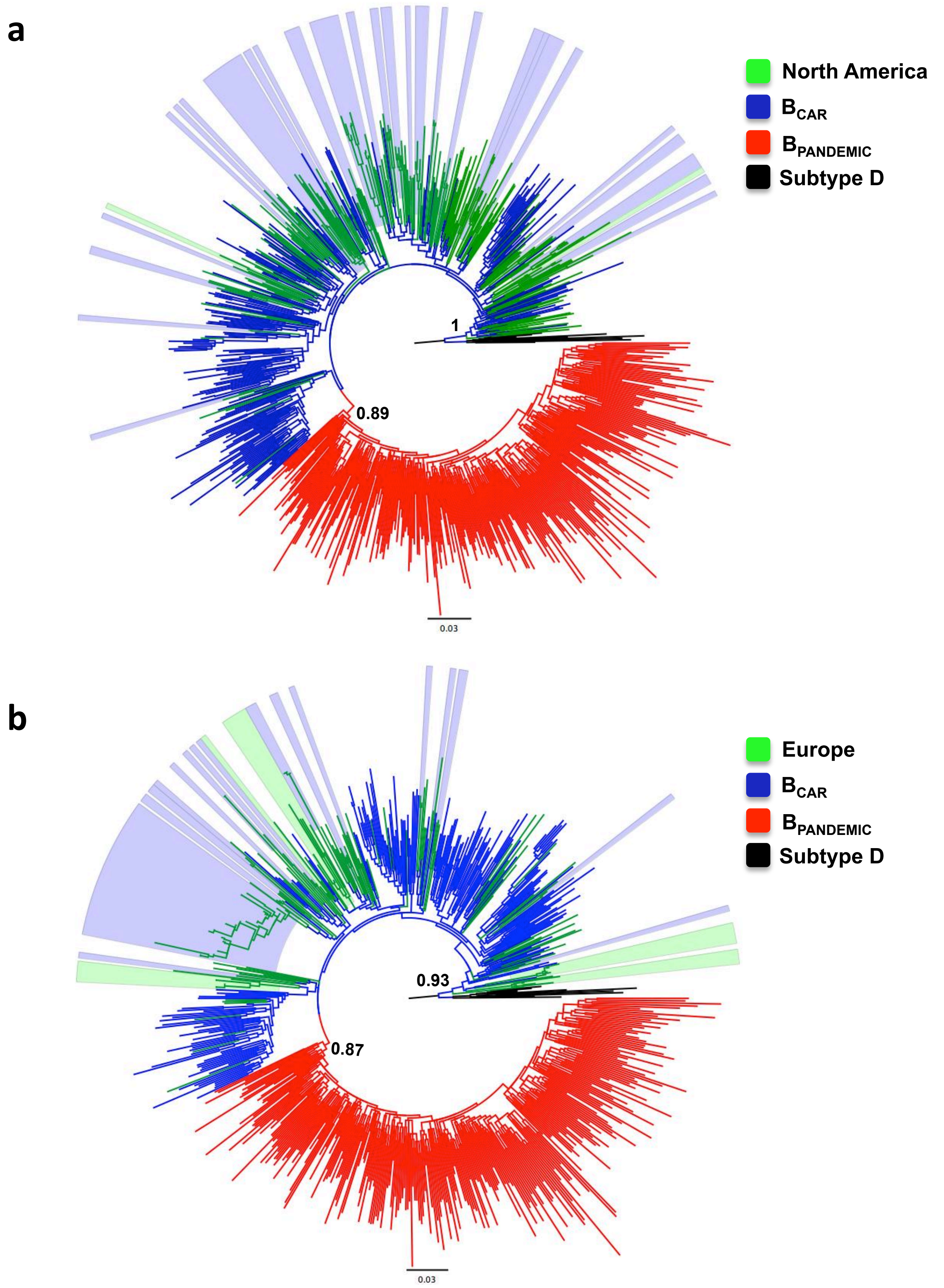

Fig. S1. **ML phylogenetic trees of HIV-1 B_{CAR} *pol* sequences identified in North America (a) and Europe (b).** North American (n = 274) and European (n = 189) B_{CAR} sequences were combined with representative sequences of the $B_{PANDEMIC}$ (US = 165, France = 135) and the B_{CAR} (Caribbean = 200) clades. Branches are colored according to the geographic origin/clade classification of each sequence as indicated at the legend (upper right). Strongly supported (*SH-aLRT* $\geq$ 0.95) country-specific (blue boxes) and international (green boxes) B_{CAR} clades from North America and Europe are indicated. SH-a*LRT* supports for subtype B and $B_{PANDEMIC}$ clades are shown. Trees were rooted and scaled as explained in Fig. 1.
